# Combined inhibition of lysine-specific demethylase 1 and kinase signaling as a preclinical treatment strategy in glioblastoma

**DOI:** 10.1093/noajnl/vdaf246

**Published:** 2025-11-20

**Authors:** Lea M Stitzlein, Deokhwa Nam, Faith A Hernandez, Kareena H Patel, Alaina Poche, Huaxian Ma, Katie Impelman, Joy Gumin, Heping Wang, Jing Wang, Samantha Gadd, Wafik Zaky, Oren Becher, Richard W Dudley, Frederick F Lang, Gangadhara R Sareddy, Joya Chandra

**Affiliations:** Department of Pediatrics, University of Texas MD Anderson Cancer Center, Houston; The University of Texas MD Anderson Cancer Center UTHealth Houston Graduate School of Biomedical Sciences, Houston; Department of Pediatrics, University of Texas MD Anderson Cancer Center, Houston; Department of Pediatrics, University of Texas MD Anderson Cancer Center, Houston; The University of Texas MD Anderson Cancer Center UTHealth Houston Graduate School of Biomedical Sciences, Houston; Department of Pediatrics, University of Texas MD Anderson Cancer Center, Houston; Department of Pediatrics, University of Texas MD Anderson Cancer Center, Houston; Department of Pediatrics, University of Texas MD Anderson Cancer Center, Houston; Department of Pediatrics, University of Texas MD Anderson Cancer Center, Houston; The University of Texas MD Anderson Cancer Center UTHealth Houston Graduate School of Biomedical Sciences, Houston; Department of Neurosurgery, University of Texas MD Anderson Cancer Center, Houston; Department of Bioinformatics and Computational Biology, University of Texas MD Anderson Cancer Center, Houston; Department of Bioinformatics and Computational Biology, University of Texas MD Anderson Cancer Center, Houston; Department of Pathology, Northwestern University, Chicago; Department of Pediatrics, University of Texas MD Anderson Cancer Center, Houston; Department of Neuro-Oncology, University of Texas MD Anderson Cancer Center, Houston; Department of Pediatrics, Icahn School of Medicine at Mount Sinai, New York; Department of Pharmaceutical Sciences, College of Pharmacy, University of Findlay, Findlay; Department of Neurosurgery, University of Texas MD Anderson Cancer Center, Houston; Department of Obstetrics and Gynecology, University of Texas Health San Antonio, San Antonio; Mays Cancer Center, University of Texas Health San Antonio, San Antonio; Department of Pediatrics, University of Texas MD Anderson Cancer Center, Houston; The University of Texas MD Anderson Cancer Center UTHealth Houston Graduate School of Biomedical Sciences, Houston; Department of Epigenetics and Molecular Carcinogenesis, University of Texas MD Anderson Cancer Center, Houston

**Keywords:** glioblastoma stem cell, kinase signaling, lysine specific demethylase-1, osimertinib

## Abstract

**Background:**

Lysine-specific demethylase 1 (LSD1) is overexpressed in glioblastoma, contributing to tumor growth and treatment resistance. LSD1 inhibitors have shown preclinical promise but have had limited clinical development for glioblastoma. Given the frequent kinase pathway alterations seen in glioblastoma, the interplay between LSD1 inhibition and kinase signaling pathways was investigated.

**Methods:**

Glioblastoma stem cell (GSC) lines and normal human astrocytes (NHAs) were treated with catalytic LSD1 inhibitors, NCD38 and bomedemstat, and the LSD1 scaffolding inhibitor, seclidemstat alone and in combination with kinase inhibitors, including osimertinib, afatinib, and ulixertinib. The effect on cell viability, proliferation, and neurosphere formation was assessed, and synergy scores were calculated using Bliss synergy models. Kinase signaling was analyzed and *in vivo* efficacy was evaluated in orthotopic xenograft models.

**Results:**

LSD1 knockdown and seclidemstat reduced kinase signaling, while catalytic LSD1 inhibitors increased kinase activity or had no effect. Catalytic LSD1 inhibitors combined with kinase inhibitors, synergistically reduced GSC viability and proliferation while sparing NHAs. Combination treatment consistently reduced phospho-S6 ribosomal protein levels in three different GSC lines, and basal phospho-S6 ribosomal protein levels across the GSCs and the NHAs were negatively correlated with a synergistic response. The generation of an NCD38-resistant GSC showed increased kinase activity and was associated with enhanced osimertinib sensitivity. Combined treatment with NCD38 and osimertinib in glioblastoma-bearing mice delayed tumor growth and improved survival outcomes.

**Discussion:**

These findings provide a rationale for further investigation of combination therapies of catalytic inhibitors of LSD1 and EGFR and dual-targeted inhibitors to overcome resistance and improve outcomes.

Key Points

- Knockdown of the histone demethylase, LSD1, reduced kinase signaling in glioblastoma stem cell models, demonstrating LSD1 regulation of kinase activation.
- Combined inhibition of LSD1 and EGFR showed additive effects *in vitro* and *in vivo*.

Importance of the Study

This study investigates the effect of lysine-specific demethylase 1 (LSD1) inhibitors in glioblastoma models to address an ongoing need for improved therapeutic options. Building upon prior studies that utilize LSD1 inhibitors as a monotherapy in brain tumor models, our work explores a rationally designed combination treatment strategy to target both LSD1 and aberrant kinase signaling pathways frequently altered in glioblastoma. This treatment combination allows for dose reductions while preserving an antitumor effect in *in vitro* and *in vivo* models. As LSD1 inhibitors continue to progress in clinical development for many cancer types, our work has highlighted the potential to further enhance their efficacy through combination treatment regimens. Importantly, this work supports studies using LSD1-based combination therapies with an overarching goal to expand treatment options for patients with glioblastoma, a population with historically limited treatment options.

Glioblastoma is an aggressive brain tumor associated with an extremely poor prognosis, with a five-year survival of only 5%-7%.^1–4^ Treatment options remain limited due to minimal advances in approved therapies over recent years, underscoring the need for novel therapeutic approaches.^5^ Lysine-specific demethylase 1 (LSD1/KDM1A) is a histone demethylase that has been identified as a potential therapeutic target in HGG, including adult glioblastoma. Through its regulation of gene expression, LSD1 can promote tumor growth and metastasis and contribute to drug resistance.^6^^,^^7^ In glioblastoma, LSD1 is overexpressed and functions to keep the cells in a stem-like state and inhibit differentiation.^8^^,^^9^

The growing interest in LSD1 as a potential therapeutic target in cancer has stimulated the development of various small-molecule inhibitors impacting different functions of LSD1. LSD1 functions as both an enzymatic demethylase to remove methyl groups from lysine residues and as a scaffolding protein to facilitate the recruitment of co-regulatory complexes. These functions of LSD1 have inspired the discovery of catalytic LSD1 inhibitors capable of inhibiting the demethylase activity of LSD1 and include molecules such as tranylcypromine, bomedemstat, NCD38, and GSK-LSD1, among others.^10–13^ Previous studies in our laboratory and that of others have demonstrated the preclinical utility of varied LSD1 inhibitors, such as NCD38, GSK-LSD1, DDP-38003, and S2172.^8^^,^^9^^,^^14–16^ Inhibition of LSD1 with the aforementioned small molecules afforded reduced tumor growth and improved survival in glioblastoma models.^8^^,^^9^^,^^14–16^ The other class of LSD1 inhibitors, scaffolding inhibitors such as seclidemstat, prevent LSD1’s ability to assemble epigenetic complexes.^17^ One concern with LSD1 inhibitors and epigenetically directed therapies in general is their effect on normal cells. Despite LSD1 being overexpressed in glioblastoma, other tissue types may retain LSD1 expression, leading to potential toxicities under therapeutic inhibition. To mitigate toxicities and improve tumor selectivity, one strategy is to use LSD1 inhibitors as part of a combination treatment strategy, allowing for dose reductions while maintaining antitumor effects. This strategy shows promise in preclinical leukemia models, as an LSD1 inhibitor-based combination treatment strategy in conjunction with WNT pathway inhibition has been shown to reduce tumor burden and extend survival.^18^ The specific LSD1 inhibitor utilized in this recently published study was GSK-LSD1, which is not clinically viable but is a valuable tool compound. Importantly, the development of LSD1 inhibitor-based combination treatment strategies for brain tumors needs to prioritize brain penetrance and limited toxicity to non-transformed neuronal cells with enhanced efficacy as an adjunct treatment.

In addition to epigenetic dysregulation, several kinase alterations are commonly observed in glioblastoma. For example, more than half of patients with glioblastoma have tumors harboring alterations in the epidermal growth factor receptor (EGFR), such as amplification or mutation.^19^^,^^20^ The phosphatidylinositol 3-kinase (PI3K) pathway is frequently hyperactivated through activating mutations in *PIK3CA* or loss-of-function mutations in *PTEN* (phosphatase and tensin homolog).^21^^,^^22^ Alterations are also commonly found in the MEK/ERK (mitogen-activated protein kinase/extracellular signal-regulated kinase) pathway and in the cyclin-dependent kinase (CDK) cell cycle regulation pathways, which can contribute to glioblastoma development and proliferation.^23^^,^^24^

Considering the existing research, which highlights the interplay between LSD1 and various kinase signaling pathways in several cancer models, including PI3K/AKT/mTOR and MEK/ERK,^25–33^ our laboratory sought to understand how LSD1 and its inhibition influence the kinase signaling pathways critical in the regulation of glioblastoma growth and survival. Given the underlying molecular alterations in glioblastoma, such an investigation is highly relevant and urgently needed. Studies have shown that LSD1 and its inhibition can impact key components of these signaling pathways, thereby influencing cell proliferation, cell growth, and cell death.^25–33^ Understanding these interactions in the context of glioblastoma will provide valuable insights to guide the development of LSD1 inhibitors as part of a therapeutic strategy.

In this study, we show that inhibition of LSD1 activity can modulate proliferative kinase signaling pathways, PI3K/AKT/mTOR and MEK/ERK. This effect varies across the LSD1 inhibitors, with reduced kinase signaling observed from LSD1 knockdown and seclidemstat. In contrast, the catalytic LSD1 inhibitors either had no effect or increased kinase activity after short-term drug exposure. Interestingly, we found that the addition of kinase pathway inhibitors targeting EGFR and MAPK, to catalytic LSD1 inhibitors, synergistically reduced glioblastoma viability. This effect was much more pronounced with the catalytic LSD1 inhibitors compared with the scaffolding inhibitor, seclidemstat. Furthermore, the combination of the catalytic LSD1 inhibitor NCD38 and osimertinib (EGFR inhibitor) was found to reduce cell proliferation and neurosphere formation. We identified that the level of phospho-S6-ribosomal protein was consistently correlated with a response to the combination treatment and its activity was increased with the generation of an NCD38-resistant cell line. Finally, the combination of NCD38 and osimertinib was found to reduce tumor burden and improve overall survival in orthotopic xenograft models of glioblastoma. In summary, our study contributes to the understanding of the interplay between LSD1 and kinase signaling pathways and provides a rationale for exploring combined treatment approaches in glioblastoma.

## Methods

### Cell Lines

The MDA-GSC lines, including the MDA-GSC17 (RRID: CVCL_DR57), MDA-GSC6-27, MDA-GSC20, MDA-GSC7-11, and MDA-GSC8-11(RRID: CVCL_DR60) were developed from patients with glioblastoma in accordance with the Declaration of Helsinki. These lines were established with informed consent and approved by the University of Texas MD Anderson Institutional Review Board under protocol LAB 04-0001 led by Dr Frederick Lang, as previously described.^8^ Immortalized normal human astrocytes (NHA/E6/E7/Tert) (RRID: CVCL_E3G4) were kindly provided by Dr Russell Piper. The MDA-GSCs and NHA were grown as previously described.^8^ All cell lines were authenticated every 6 months using the Cytogenetics and Cell Authentication Core at The University of Texas MD Anderson Cancer Center (RRID: SCR_026262).

### Reagents

Seclidemstat (CAS# 1423715-37-0), GSK-LSD1 (CAS# 2102933-95-7), osimertinib (CAS# 1421373-65-0), ulixertinib (CAS# 869886-67-9), and afatinib (CAS# 850140-73-7) were purchased from MedChemExpress (Monmouth Junction, NJ). NCD38 was custom synthesized with New Business Development Co., Ltd (Tokyo, Japan) and bomedemstat (CAS #1990504-72-7) was kindly provided by Merck. All inhibitors were diluted to 10 or 25 mM with dimethyl sulfoxide (DMSO) according to the manufacturer’s instructions and stored at −80 °C.

### AlamarBlue Cell Viability Assay

The cell lines were plated at 10,000 cells/well into a 96-well plate and allowed to grow for 2–3 days before treatment. After 72 hours of treatment, the AlamarBlue reagent (Bio-Rad; Hercules, CA) was added to each well and incubated according to the manufacturer’s protocol. Fluorescence, an indication of cell viability, was measured with the Synergy 2 (BioTek Instruments; Winooski, VT) (RRID: SCR_019765) plate reader.

### Western Blot Analysis

Protein was extracted from cultured cells using RIPA lysis buffer (10 mM Tris-HCl, 1 mM EDTA, 0.5 mM EGTA, 1% Triton X-100, 0.1% Sodium Deoxycholate, 0.1% SDS, 140 mM NaCl) supplemented with protease and phosphatase inhibitors (Roche; Basel, Switzerland). After the Bradford assay, protein concentration was adjusted to 2.5 µg/µl, and 50 µg of protein was loaded per well into 10% polyacrylamide gels. Following electrophoretic separation, the protein was transferred to a nitrocellulose membrane and blocked with 10% milk for 1 hour at room temperature. Primary antibodies were diluted in 5% BSA and incubated with the membrane at 4 °C overnight. The membrane was incubated in secondary antibodies for 1 hour at room temperature before imaging the membrane with ECL reagent (Cell Signaling Technologies; Danvers, MA) using the ChemiDoc (Bio-Rad; Hercules, CA) (RRID: SCR_019037) and analyzed using Image Lab (Bio-Rad; Hercules, CA) (RRID: SCR_014210). Primary antibodies were purchased from Cell Signaling Technologies (Danvers, MA) (phospho-ERK1/2 (# 9101, RRID: AB_331646), ERK1/2 (# 9102; RRID: AB_330744), phospho-AKT (# 4058; RRID: AB_331168), AKT (# 9272; RRID: AB_329827), phospho-S6 Ribosomal Protein (# 4857; RRID: AB_2181035), S6 ribosomal protein (# 2217; RRID: AB_331355), LSD1 (# 2184; RRID: AB_2070132), Actin (# 3700; RRID: AB_2242334), Tubulin (# 5568; RRID: AB_10694505), and GAPDH (# 5174; RRID: AB_10622025)). Secondary HRP-linked antibodies were also purchased at Cell Signaling Technologies (# 7074 and 7076; RRID: AB_2099233 and RRID: AB_330924). Please see the Supplementary Material for raw western images.

### LSD1 Knockdown

Lentiviral vectors were produced by co-transfecting HEK293T cells (RRID: CVCL_0063) with LSD1 shRNA plasmid (RHS4430-200165880 Clone ID: V2LHS_34924(ORF)ORF; Mature antisense: AAAGGTTTGACTCGTGGAG), psPAX2 (Addgene; Watertown, MA) (RRID: Addgene_12260), and PMD2.G (Addgene; Watertown, MA) (RRID: Addgene_12259) using JetPrime transfection reagent (Polyplus Transfection; Graffenstaden, France). After 2 days, the virus was collected from the supernatant and concentrated. MDA-GSC17 cells were plated at 0.5 × 10^6^ cells per well into 6-well plates. After 48 hours, the concentrated lentivirus was added along with 10 µg/ml polybrene (Merck KGaA; Darmstadt, Germany). Media was replaced after 2 days, and transduced cells were selected with 2.5 µg/ml puromycin (Selleckchem; Houston, TX) for 1-2 weeks. Gene knockdown was evaluated via Western blot analysis.

### Synergy Calculation

Synergy scores were calculated using SynergyFinder.com.^34^ The normalized data from the alamarBlue assays were uploaded as percent viability. The Bliss synergy score was used to determine the drug combination’s relationship, where a score greater than 10 indicates a synergistic interaction, a score between −10 and 10 indicates an additive effect, and less than −10 indicates an antagonistic interaction.

### Vi-Cell Proliferation Assay

The MDA-GSCs and NHA cell lines were plated at 1 × 10^6^ cells into T25 flasks and allowed to grow for 1-2 days. The cells were then treated with either DMSO (0.5%), NCD38 (3 µM), osimertinib (5 µM), or a combination of NCD38 (3 µM) and osimertinib (5 µM) for 48 hours. Cell viability was determined with the Vi-Cell XR Cell Viability Analyzer (Beckman Coulter; Brea, CA) (RRID: SCR_019664) using trypan blue as the viability dye.

### Neurosphere Formation Assay

Neurosphere formation assay was performed on MDA-GSCs using clear, flat-bottom 96-well plates. The cells were plated at 1,000 cells/well into 100 µl of media. Immediately following plating, the MDA-GSCs were treated with either NCD38, osimertinib, or a combination of NCD38 and osimertinib. After one week, the plates were imaged using the Nikon Eclipse Ti microscope (Nikon; Tokyo, Japan) (RRID: SCR_021242) and counted using ImageJ (RRID: SCR_003070) to obtain the average number of neurospheres that formed from each treatment condition.

### Reverse Phase Protein Array (RPPA) Analysis

Protein was extracted from brain tissue using RPPA lysis buffer (1% Triton X‐100, 50 mM HEPES pH 7.4, 150 mM NaCl, 1.5 mM MgCl2,1 mM EGTA, 100 mM NaF, 10 mM Na pyrophosphate, 1 mM Na3VO4, 10% glycerol) and adjusted to 1.5 μg/μl. Protein was denatured with 4X SDS and 2-mercaptoethanol, followed by 5 minutes of heat at 95°C. The RPPA was performed by the University of Texas MD Anderson Cancer Center RPPA Core facility (RRID: SCR_016649) as previously described^35^^,^^36^ and analyzed with R (RRID: SCR_001905). The relative protein expression data (Level 4 (Log_2)) were analyzed using one-way ANOVA, followed by Tukey’s post hoc multiple comparisons test to identify differentially expressed proteins. A heatmap was generated to visualize the expression levels of differentially expressed proteins across samples.

### Generation of an NCD38-Resistant Cell Line

MDA-GSC17 cells were cultured with increasing concentrations of NCD38 over the span of 2-3 months. On the first day, the cells were treated with a non-toxic dose of NCD38 (2 µM). After every four or five passages, or when the cell viability and cell count were not affected by NCD38, the concentration was increased by 1 µM until the concentration reached 4 µM. The MDA-GSC17 cells were considered moderately resistant to NCD38 when the cells treated with NCD38 (4 µM) had no change in cell viability or cell count compared with untreated control.

### In Vivo Orthotopic Tumor Model

Mice (athymic nude female; 8-10 weeks of age) (The Jackson Laboratory; Bar Harbor, ME) (RRID: IMSR_JAX: 002019) were bolted intracranially as previously described with a low mortality rate from the procedure and implanted with 500,000 MDA-GSC17 luciferase-expressing cells 1 week later via guide screw.^37^ After confirming tumor engraftment 48 hours after implantation via In Vivo Imaging System (IVIS) luminescent imaging (Capilar Life Sciences; Hopkinton, MA), mice were separated into groups (*n* = 4 or 5 mice) to receive either vehicle control, NCD38 10 mg/kg, osimertinib 50 mg/kg, or a combination of NCD38 and osimertinib. Distribution of the mice was based on IVIS imaging data to ensure an equal average tumor volume across each group before treatment began. NCD38 was prepared in a solution of 30% Captisol (CyDex Pharmaceuticals; Lenexa, KS) in water. Osimertinib was prepared in 0.4% between 80 and 0.5% methylcellulose in water. The drugs were administered to the mice daily via oral gavage until they succumbed to tumor burden or reached a humane endpoint (moribund, seizure, weight loss >20%). Mice were imaged weekly by injecting luciferin (4 mg/mouse) subcutaneously. After 10 minutes, luminescent images were acquired and analyzed with total flux. Weight was measured weekly and compared between vehicle-treated control and experimental conditions. Blood parameters were determined using a CBC Hematology Analyzer for veterinary samples. All animal experiments were approved by the University of Texas MD Anderson Cancer Center Institutional Animal Care and Use Committee (IACUC) under protocol 0000638 (P.I. Chandra).

### Cellular Thermal Shift Assay (CETSA)

Brain tissues were resuspended in CETSA buffer (10 ml PBS with Roche complete ULTRA protease inhibitor cocktail tablet (Roche; Basel, Switzerland)). The samples, approximately 50 µg per condition, were incubated at increasing temperatures (42, 44, 48, and 52 °C) to induce protein denaturation and precipitation of proteins that are not bound to a ligand. Following exposure to heat, the samples were lysed with three freeze/thaw cycles using liquid nitrogen. Finally, the soluble protein fraction was collected and examined using Western blot analysis to detect the stability of LSD1 in the control mice compared with NCD38-treated mice.

### Statistical Analysis

The difference between vehicle-treated control and experimental conditions was evaluated using a student’s *t*-test, one-way ANOVA, two-way ANOVA, or Log-rank test performed with GraphPad Prism 10 software (RRID: SCR_002798).

## Results

### Effect of LSD1 Inhibition on Kinase Signaling

**Figure 1.**
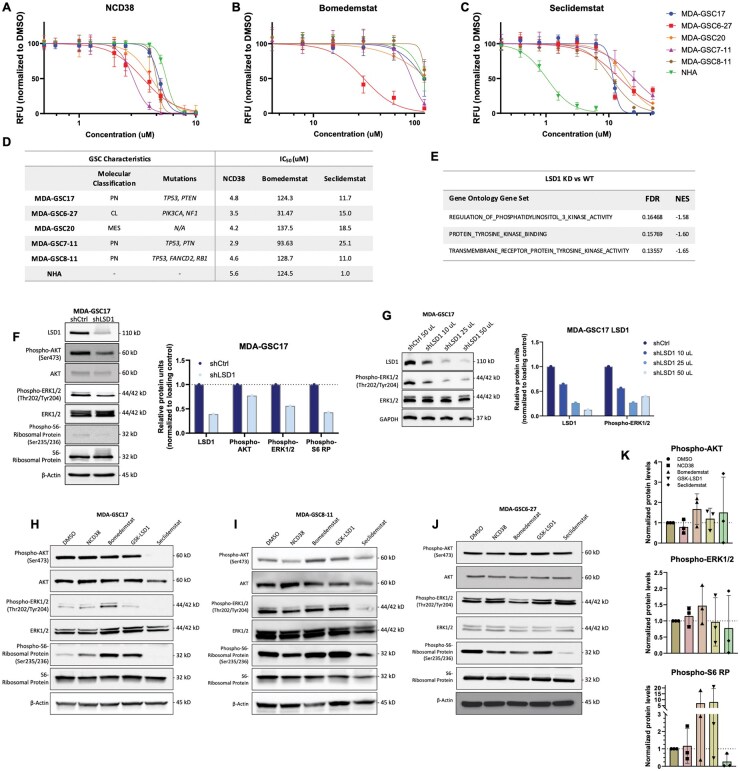
**Effect of LSD1 inhibition on kinase signaling pathways in GSCs**. Cell viability of MDA-GSCs and NHAs treated with (A) NCD38, (B) bomedemstat, or (C) seclidemstat after 72 hours. Dose responses were replicated in triplicate and are shown as the mean with standard deviation. (D) IC_50_ chart of the single-agent LSD1 inhibitors in the MDA-GSCs and NHAs and summary of GSC characteristics. (E) Kinase-related gene sets from the Gene Ontology GSEA comparing LSD1 knockdown with LSD1 wildtype in the glioblastoma cell line, LN18. Western blot comparing the (F) knockdown of LSD1 in the MDA-GSC17 with a non-targeting shRNA sequence, with quantification, and (G) a dose-dependent effect of LSD1 knockdown on ERK1/2 activity, with quantification. Western blot of the (H) MDA-GSC17s,( I) MDA-GSC8-11s, and (J) MDA-GSC6-27s treated with either DMSO, NCD38 (3 µM), bomedemstat (100 µM), GSK-LSD1 (500 µM), or seclidemstat (10 µM). Doses were chosen based on the IC_50_ across the GSC lines to ensure a sub-lethal effect that is biologically relevant. (K) Quantification of Western blots for the MDA-GSC17, −8–11, and −6–27s treated with LSD1 inhibitors. RFU, relative fluorescent units; PN, proneural; CL, classical; MES, mesenchymal; FDR, false discovery rate; NES, normalized enrichment score.

LSD1 inhibitors, either catalytic or scaffolding, can have distinct effects from one another on kinase signaling and cell viability. In this study, three LSD1 inhibitors were screened against a panel of five GSC lines and normal human astrocytes (NHAs), which serve as a non-malignant control. Two catalytic inhibitors were included in this screen, NCD38 (Figure 1A) and bomedemstat (Figure 1B), as well as one scaffolding inhibitor, seclidemstat (Figure 1C), to assess their effects on cell viability.

The GSC lines included in this panel represent distinct molecular subtypes of glioblastoma, including three proneural, one classical, and one mesenchymal, and differ in key genetic alterations as described previously.^8^ The half-maximal inhibitory concentrations (IC_50_) were calculated from the corresponding dose-response curves (Figure 1D), with NCD38 showing effects on GSCs cell viability at 3-5 µM, bomedemstat at 30-125 µM, and seclidemstat at 10-25 µM. The MDA-GSC6-27 did demonstrate increased sensitivity to bomedemstat compared with the other GSC lines, which may warrant further investigation. However, there was little selectivity of the LSD1 inhibitors to affect the GSC lines while sparing the NHAs. Gene set enrichment analysis (GSEA) was utilized from our previously published study to identify potential vulnerabilities associated with LSD1 inhibition.^8^ The comparison of LSD1 knockdown with LSD1 wild type in the glioblastoma cell line, LN18 (RRID: CVCL_0392), revealed enrichment of gene sets involving kinase signaling pathways with LSD1 expression (Figure 1E). These gene sets included genes related to receptor tyrosine kinase (RTK) activity, RTK binding, and regulation of PI3K signaling.

Given that kinase signaling pathways, particularly the PI3K/AKT and MAPK pathways, are relevant to glioblastoma, the impact of LSD1 knockdown on these signaling networks was investigated. Knockdown of LSD1 in the MDA-GSC17s showed that reduced LSD1 expression was associated with a decrease in kinase activity at phospho-AKT, phospho-ERK1/2, and phospho-S6 ribosomal protein (Figure 1F). Furthermore, LSD1 knockdown resulted in a reduction in phospho-ERK1/2 that correlated with decreasing LSD1 expression (Figure 1G). The lysates were generated from cells transduced with shRNA targeting LSD1 to produce a gradient of LSD1 knockdown and assess the relationship between phospho-ERK1/2 signaling and LSD1 depletion. To determine if pharmacological LSD1 inhibitors phenocopied the effect of LSD1 knockdown, the effect of LSD1 inhibitors on kinase signaling pathways was assessed in the MDA-GSC17s, MDA-GSC8-11s, and MDA-GSC6-27s. Interestingly, the scaffolding inhibitor, seclidemstat, had a distinct effect on kinase signaling compared with the catalytic inhibitors. In the MDA-GSC17s treatment with seclidemstat resulted in decreased phospho-ERK1/2, phospho-AKT, and phospho-S6 ribosomal protein expression (Figure 1H), an effect similar to what was observed with LSD1 knockdown. In contrast, the catalytic LSD1 inhibitors, NCD38, bomedemstat, and GSK-LSD1, had either a slight increase or no effect on phospho-ERK1/2, phospho-AKT, and phospho-S6 ribosomal protein expression. Similar to the MDA-GSC17s, seclidemstat reduced kinase activity in the MDA-GSC8-11s and GSC6-27s. In the MDA-GSC8-11s, seclidemstat reduced the expression of phospho-ERK1/2 and phospho-S6 ribosomal protein compared with the catalytic inhibitors (Figure 1I). Similarly, seclidemstat reduced kinase activity at phospho-S6 ribosomal protein in the MDA-GSC6-27 (Figure 1J). The expression of phospho-AKT and phospho-ERK1/2 was not changed in the MDA-GSC6-27 with any of the four LSD1 inhibitors that were assessed. While there is a trend when comparing seclidemstat to the catalytic LSD1 inhibition, heterogeneity in kinase activity is observed among the catalytic LSD1 inhibitors across the three GSC lines assessed (Figure 1K).

### Dual Inhibition of LSD1 and Kinase Signaling Pathways

**Figure 2.**
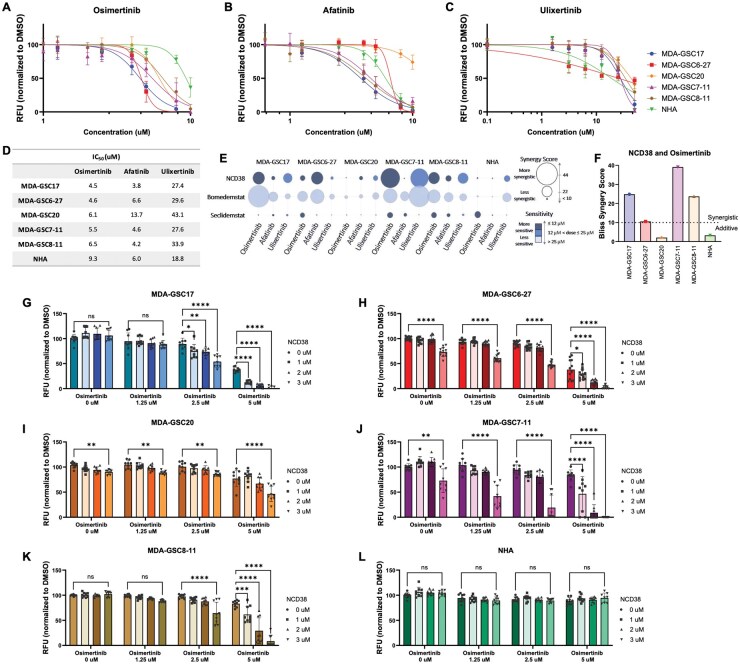
**Catalytic inhibition of LSD1 combined with kinase inhibition synergistically reduces GSC viability**. Cell viability of MDA-GSCs and NHAs treated with (A) osimertinib, (B) afatinib, or (C) ulixertinib after 72 hours. Dose responses were replicated in triplicate and are shown as the mean with standard deviation. (D) IC_50_ chart of the single-agent kinase inhibitors in the MDA-GSCs and NHAs. (E) Summary graph of the synergy scores and sensitivity for the combination of LSD1 inhibitors (NCD38 1-3 µM, bomedemstat 12.5-50 µM, or seclidemstat 3.125-12 µM) with kinase inhibitors (osimertinib 1.25-5 µM, afatinib 1.25-5 µM, or ulixertinib 6.25-25 µM) in each cell line. The size of the circles corresponds to the synergy score, ranging from 44 (the highest score) to less than 10 (a score < 10 indicates that the combination is not synergistic). The color of the circles corresponds to the dose of the drug used. (F) Bliss synergy scores for the treatment combination of NCD38 and osimertinib in the MDA-GSCs and the NHAs. Synergy scores greater than 10 indicate a synergistic relationship. Cell viability of the (G-K) MDA-GSCs and the (L) NHAs when treated with NCD38 and osimertinib for 72 hours (*n* = 3). The difference between experimental conditions was evaluated using a two-way ANOVA and Tukey’s multiple comparisons. RFU, relative fluorescent units. ns, *P* > .05; *P < .05; **P < .01; ***P < .001; ****P < .0001.

Based on the observed effect of LSD1 inhibitors on phospho-ERK1/2 and phospho-S6 ribosomal protein expression, the effects of combining LSD1 inhibitors with specific kinase inhibitors were further investigated. Since EGFR is a key regulator in glioblastoma growth and upstream of both the MAPK and PI3K/AKT pathways, inhibitors targeting EGFR and ERK1/2 were selected to assess their interactions with LSD1 inhibition. Three kinase inhibitors were selected and screened in the GSC panel and NHAs. Osimertinib (Figure 2A) and afatinib (Figure 2B) were chosen as the EGFR inhibitors based on previous literature demonstrating efficacy in glioblastoma.^38–43^ Their effects on cell viability, as determined by IC_50_ calculation, ranged from 3 to 14 µM. Ulixertinib, an ERK1/2 inhibitor, was the third kinase inhibitor and demonstrated single-agent activity at 18-43 µM (Figure 2C). The single-agent kinase inhibitor IC_50_ values were calculated from the dose-response curves and summarized according to cell line (Figure 2D). The combined effects of LSD1 inhibition and kinase inhibition on cell viability, screening nine unique treatment combinations in the GSCs and NHAs, were investigated next. This included treatment with LSD1 inhibitors, NCD38 (1-3 µM), bomedemstat (12.5-50 µM), or seclidemstat (3.125-12 µM) in combination with kinase inhibitors, osimertinib (1.25-5 µM), afatinib (1.25-5 µM), or ulixertinib (6.25-25 µM) in each cell line. The effect of combination treatment on cell viability was assessed by calculating the bliss synergy score and determining how sensitive the effects were in the GSCs and the NHAs (Figure 2E). Interestingly, the two catalytic LSD1 inhibitors, when combined with the kinase inhibitors, had a more synergistic relationship relative to the scaffolding inhibitor combined with kinase inhibitors. One GSC line, MDA-GSC20, and the NHA line largely lacked a synergistic effect from the combination treatments. The absence of a synergistic effect at the tested concentrations in the NHAs is promising as this may allow for improved selectivity with dose de-escalation.

**Figure 3.**
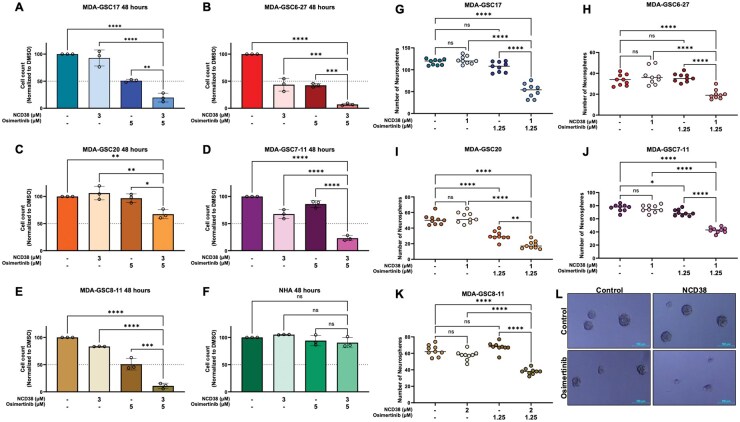
**NCD38 in combination with osimertinib reduces GSC proliferation and neurosphere formation**. (A-E) MDA-GSC and (F) NHA proliferation when treated with either NCD38, osimertinib, or a combination of NCD38 and osimertinib for 48 hours. (G-K) MDA-GSC neurosphere formation when treated with either NCD38, osimertinib, or a combination of NCD38 and osimertinib for 1 week. All experiments were replicated in triplicate and are shown as the mean with standard deviation. Differences in treatment conditions were evaluated using a one-way ANOVA with Tukey’s multiple comparisons. (L) Representative image of the MDA-GSC7-11s for counting the number of neurospheres after 1 week. ns, *P* > .05; *P < .05; **P < .01; ***P < .001; ****P < .0001.

The bliss synergy scores for the combination of NCD38 and osimertinib are compared across the GSC lines and the NHAs to further highlight the improved selectivity (Figure 2F). This treatment combination was chosen to study further due to its synergistic effects in four of the five GSC lines (Figure 2G-K) and lack of synergistic effects on NHA cell viability (Figure 2L). To validate the effect of the combination treatment on cell viability, the impact of NCD38 and osimertinib on cell proliferation and neurosphere formation was evaluated. As expected, a significant reduction in GSC proliferation from the combination treatment was observed when compared with vehicle control, single-agent NCD38, and single-agent osimertinib (Figure 3A-E). Importantly, neither the single-agent treatments nor the combination treatment influenced NHA cell proliferation (Figure 3F). The effects on proliferation were consistent with what was observed from the cell viability assays. Furthermore, GSC neurosphere formation was reduced from the combination treatment relative to vehicle control or single-agent treatments (Figure 3G-L). Overall, the combination of NCD38 and osimertinib has a synergistic effect in the GSCs while largely sparing the NHA. This synergistic effect on the GSCs is conserved across cell viability, cell proliferation, and neurosphere formation.

### Kinase Activity Correlates with Synergy and NCD38 Resistance

**Figure 4.**
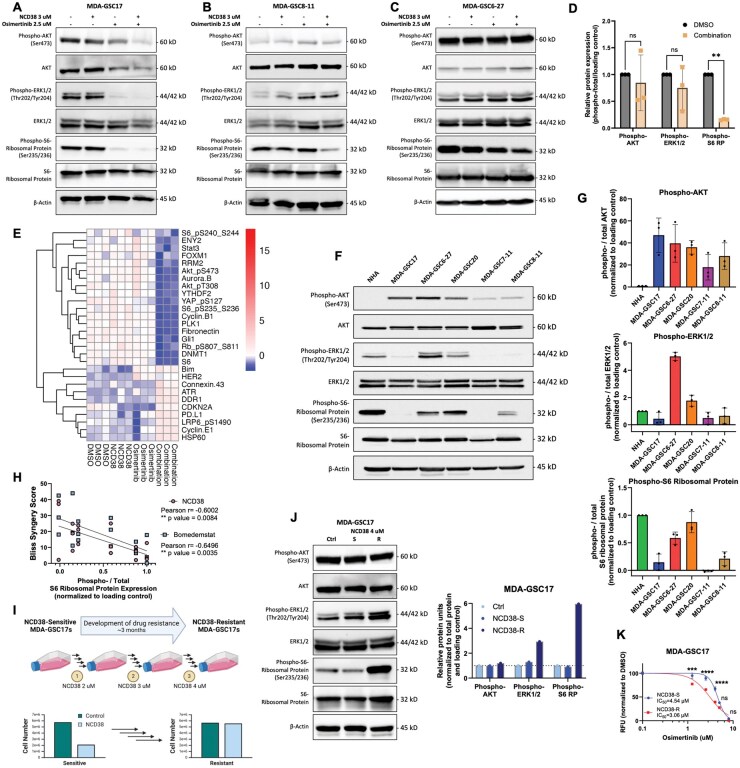
**Correlation of kinase activity with combined treatment of NCD38 and osimertinib, and with an NCD38 resistance model**. Western blot of the (A) MDA-GSC17s, (B) MDA-GSC8-11s, and (C) MDA-GSC6-27s treated with either DMSO, NCD38, osimertinib, or a combination of NCD38 and osimertinib after 24 hours (*n* = 1 each). (D) Densitometry of Western blot analysis to compare the combined effect of NCD38 and osimertinib on phospho-protein levels with untreated control in the MDA-GSC17, MDA-GSC8-11, and MDA-GSC6-27 lines. Treatment differences were evaluated using a two-way ANOVA with Sidak multiple comparisons. (E) Heatmap of differentially expressed proteins from RPPA analysis across treatment samples in the MDA-GSC17s. (F) Representative Western blot (*n* = 3) showing the kinase activity of the MDA-GSCs and NHA at baseline, without any treatment. Quantification of the baseline (G) AKT activity, ERK1/2 activity, and S6-ribosomal protein activity across the MDA-GSCs and NHA. (H) Scatter plot assessing the correlation between baseline S6-ribosomal protein activity and synergistic response to the combination treatment of NCD38 and osimertinib, as well as bomedemstat and osimertinib. Correlation was assessed using a simple linear regression to generate a Pearson correlation coefficient. There was no significant correlation between phospho-AKT and phospho-ERK1/2 levels and the synergistic response; therefore, the data are not shown. (I) Scheme describing the generation of an NCD38-resistant cell line over several weeks (created with BioRender.com), with a comparison of the NCD38-resistant cells to NCD38-sensitive cells and the effect of NCD38 on cell proliferation. (J) Western blot (*n* = 1) to compare the kinase activity of the MDA-GSC17s (baseline) with a 48-hour exposure to NCD38 treatment and the NCD38-resistant cells with quantification. (K) Single-agent dose response of osimertinib in the NCD38-sensitive and NCD38-resistant MDA-GSC17s. Differences in the effect of treatment at each dose were evaluated with a Student’s t-test. ns, *P* > .05; *P < .05; **P < .01; ***P < .001; ****P < .0001.

The combination of NCD38 and osimertinib was further assessed to determine the effect of dual inhibition on kinase signaling. In the MDA-GSC17s, there were reduced levels of phospho-AKT, phospho-ERK1/2, and phospho-S6 ribosomal protein from the combination of NCD38 and osimertinib (Figure 4A). Similarly, there were reduced phospho-S6 ribosomal protein levels in the MDA-GSC8-11 and MDA-GSC6-27s from combination treatment (Figure 4B and C). In comparison, there was no reduction in phospho-AKT or phospho-ERK1/2 in the MDA-GSC8-11 or the MDA-GSC6-27 lines. Importantly, across the three GSC lines, the combination treatment consistently reduced phospho-S6 ribosomal protein levels (Figure 4D). Furthermore, there was no significant change in total protein expression of the kinases in any of the three GSC lines. Among the kinases that were assessed, the phospho-S6 ribosomal protein level was the only one that consistently had reduced activity from the combination treatment in all three cell lines. In support of these findings, reverse phase protein array (RPPA) analysis of the MDA-GSC17 cell line treated with either a single agent or a combination of NCD38 and osimertinib, reveals a distinct effect on protein expression from the combination treatment (Figure 4E). This effect included reduced phospho-protein levels, such as phospho-AKT and phospho-S6 ribosomal proteins.

To establish whether basal kinase activity was a determinant of sensitivity to the combination treatment of LSD1 inhibitors and osimertinib, baseline kinase activity was assessed across the GSC lines and with the NHA (Figure 4F). Interestingly, phospho-AKT levels were increased in the GSC lines compared with the NHA (Figure 4G), but it did not correlate to the efficacy of the combination. Phospho-ERK1/2 levels varied across the cell lines (Figure 4G), and there was no consistent trend between phospho-ERK1/2 and the synergy of LSD1 inhibition and osimertinib. Similarly, phospho-S6 ribosomal protein levels varied across the cell lines (Figure 4G); however, these levels negatively correlated with the effectiveness of combination treatment (Figure 4H). For example, the cell lines with the highest basal phospho-S6 ribosomal protein levels (NHA and MDA-GSC20s) are the cell lines with the lowest synergy scores. In contrast, the three cell lines with the lowest phospho-S6 ribosomal protein expression (MDA-GSC17, MDA-GSC7-11, and MDA-GSC8-11) are the cell lines with the highest synergy scores from the combination treatment. The negative correlation between phospho-S6 ribosomal protein expression and synergy scores is seen for the combination of NCD38 and osimertinib as well as bomedemstat and osimertinib.

Building upon the negative correlation between phospho-S6 ribosomal protein and synergistic treatment combinations, potential underlying molecular mechanisms were investigated. To explore this relationship, an NCD38-resistant cell line was generated using the MDA-GSC17s. The NCD38-resistant MDA-GSC17s were considered resistant when there was no effect of NCD38 on cell proliferation compared to untreated control (Figure 4I). Next, the kinase activity of the MDA-GSC17s was compared across an untreated control, 48-hour NCD38 treatment, and the NCD38-resistant MDA-GSC17s. Interestingly, there was an upregulation of kinase activity at phospho-ERK1/2 and phospho-S6 ribosomal protein in the NCD38-resistant line compared to untreated control and 48-hour NCD38 exposure (Figure 4J). There was no difference in phospho-AKT observed across these treatment conditions. The upregulation of kinase activity suggests a compensatory response to LSD1 inhibition that may contribute to the synergistic relationship between NCD38 and osimertinib. Furthermore, the NCD38-resistant line was compared with the parental MDA-GSC17 based on the response to single-agent osimertinib treatment. The NCD38-resistant line had enhanced sensitivity to osimertinib relative to the parental MDA-GSC17s (Figure 4K).

### Combined Inhibition of LSD1 and EGFR in an Orthotopic Xenograft Model of Glioblastoma

**Figure 5.**
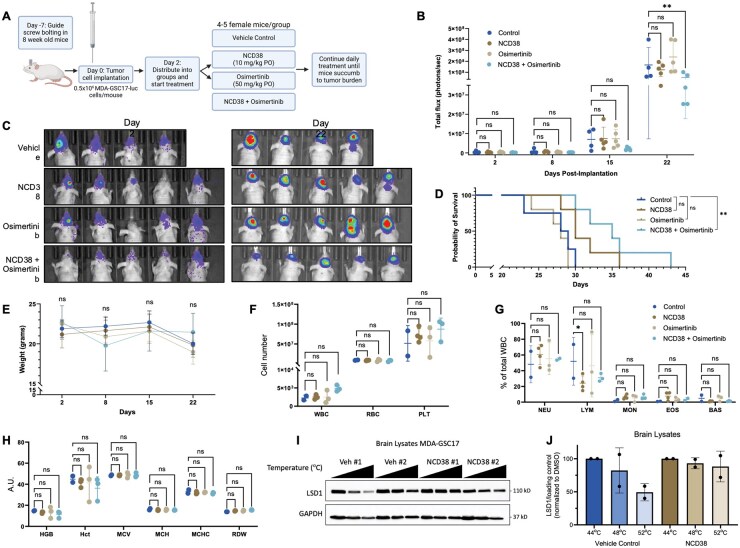
**Combined treatment of NCD38 and osimertinib improves efficacy in orthotopic xenograft models of glioblastoma**. (A) Timeline of the *in vivo* experiment and dosing regimen (created with BioRender.com). (B) Tumor burden measured by total flux (photons/second) at baseline (day 2 post-implantation) and after 1, 2, and 3 weeks of daily dosing. (C) Luminescent images of the mice orthotopically implanted with the MDA-GSC17-luc cells prior to the start of treatment and after 3 weeks of treatment. (D) Overall survival curve of the mice to compare vehicle control with the single-agent treatment groups and the combination treatment group. Survival distribution was compared using a log-rank test. (E) Weight of the mice prior to treatment and after 1, 2, and 3 weeks of treatment. At the end of the study, blood samples were collected from each mouse and analyzed for (F) complete blood counts, (G) white blood cell (WBC) differential, and (H) red blood cell (RBC) parameters. Differences in treatment conditions were evaluated using a two-way ANOVA with Tukey’s multiple comparisons. (I and J). Thermal stability of LSD1 in NCD38-treated mice (*n* = 2) compared with vehicle control-treated mice (*n* = 2) assessed by a cellular thermal shift assay (CETSA). ns, *P* > .05; *P < .05; **P < .01; ***P < .001; ****P < .0001. WBC, white blood cell; RBC, red blood cell; PLT, Platelet; NEU, neutrophils; LYM, lymphocytes; MON, monocytes; EOS, eosinophils; BAS, basophils; HGB, hemoglobin; Hct, hematocrit; MCV, mean corpuscular volume; MCH, mean corpuscular hemoglobin; MCHC, mean corpuscular hemoglobin concentration; RDW, red cell distribution width.

The demonstrated synergistic efficacy (cell viability, cell proliferation, and neurosphere formation) seen *in vitro* for combined LSD1 and EGFR inhibition prompted an *in vivo* evaluation of NCD38 and osimertinib using an orthotopic glioblastoma xenograft mouse model. Mice were intracranially injected with luciferase-labeled MDA-GSC17 cells (MDA-GSC17-luc) and randomized into treatment groups to evaluate the therapeutic response, including tumor growth and survival monitoring (Figure 5A). Two days after tumor implantation via guide screw, engraftment was confirmed with luminescent imaging and the mice began receiving daily treatment of either vehicle control, NCD38, osimertinib, or a combination of NCD38 and osimertinib. Tumor burden was monitored through luminescent imaging each week. After 3 weeks of treatment, a significant reduction in luminescence signal from the mice receiving the combination treatment of NCD38 and osimertinib compared with the vehicle control was observed (Figure 5B and C). At this same time point, the mean luminescence signal of the control mice was more than double that of the combination treatment group (1.68 × 10^8^ photons/second in the control mice compared with 5.15 × 10^7^ photons/second in the combination mice). Corroborating the difference in luminescence signal as a measure of efficacy was the survival of the mice in the combination treatment cohort compared to vehicle control (Figure 5D). Here, mice receiving the combination treatment of NCD38 and osimertinib survived approximately 1 week longer than the control mice (*P *= .0072), with a median survival of 35 days compared with 28.5 days. This improvement in survival was unique to the combination treatment group, as neither NCD38 nor osimertinib as monotherapy demonstrated a significant survival benefit compared with the control mice.

Nonclinical toxicology was assessed in addition to the efficacy of the NCD38 and osimertinib combination in the described glioblastoma mouse model. Mice in all treatment groups were weighed weekly to monitor for weight loss or signs of toxicity associated with the treatment. No differences in weight were observed across the treatment groups (Figure 5E). Hematological parameters also revealed no important difference across the treatment groups (Figure 5F-H). Importantly, maintenance of weight and hematological status were maintained after several weeks of daily dosing, indicating no observable or overt toxicity from the treatment combination impacting the overall health of the mice.

Osimertinib is well established as a brain-penetrant EGFR inhibitor; however, data for NCD38 is more limited. Using brain protein lysates from NCD38-treated mice and matched controls, a cellular thermal shift assay (CETSA) demonstrated enhanced stability of LSD1 in the treatment group relative to vehicle control (Figure 5I and J). This finding supports the brain-penetrant physicochemical properties of NCD38.

## Discussion

Numerous LSD1 inhibitors have been designed and are in development, yet clinical progress for the treatment of solid tumors, particularly brain tumors, remains limited. Several factors in preclinical studies may contribute to this lack of advancement, including toxicity, poor brain penetrance, limited efficacy at the tumor site, and resistance. A barrier to effective LSD1-targeted therapies is the development of resistance, where tumor cells adapt by activating compensatory signaling to enhance survival mechanisms. In this study, we generated an NCD38-resistant GSC line and observed kinase pathway activation, particularly in the PI3K/AKT and MAPK/ERK cascades. Our previous transcriptomic analyses suggest changes in kinase-related gene expression and reinforce that glioblastoma cells may evade LSD1 inhibition by upregulating kinase signaling and contributing to resistance.^8^ Understanding adaptive responses to LSD1 inhibitors is critical for the development of more effective therapeutic strategies that can overcome potential resistance mechanisms. Our study addresses these challenges by investigating the effects of LSD1 inhibition on proliferative kinase signaling pathways, including PI3K/AKT/mTOR and MEK/ERK, and identifying strategies to enhance the efficacy of small-molecule LSD1 inhibitors through combination treatment.

Interestingly, inhibiting different functions of LSD1, targeting its catalytic amine oxidase domain versus disrupting its scaffolding function, or LSD1 knockdown, leads to differential effects on kinase signaling pathways. Where seclidemstat is similar to LSD1 knockdown and reduces the activity of kinase signaling, catalytic inhibition can be associated with an increase in kinase activity at phospho-ERK1/2 and phospho-S6 ribosomal protein. Consequently, our study revealed that catalytic LSD1 inhibitors, and not seclidemstat, have a synergistic relationship with kinase inhibitors targeting the EGFR and ERK1/2 pathway in several of the GSC lines. NCD38 and osimertinib were further investigated to demonstrate that combined inhibition of LSD1 and EGFR effectively reduced GSC cell viability, cell proliferation, and neurosphere formation in culture. When assessed in glioblastoma-bearing mouse models, the combination of NCD38 and osimertinib demonstrated a reduction in tumor burden, as evidenced by a significant reduction in tumor luminescence signal and improved survival rates. Overall, this study contributed to identifying the contrasting effects of LSD1 inhibitors and the utility of combination treatment in the context of catalytic LSD1 inhibition.

Our study builds on previous research evaluating targeted therapies against LSD1 or EGFR in glioblastoma, with both similarities and differences in efficacy. Prior investigations have assessed the efficacy of LSD1 inhibitors, including NCD38, in glioblastoma models with promising results.^8^^,^^9^^,^^14^^,^^15^^,^^44^^,^^45^ While our *in vitro* data is comparable in terms of NCD38 efficacy, the *in vivo* monotherapy is not as strong.^9^^,^^14^ As a single agent, NCD38 did not provide a significant survival benefit, but the data exhibited a trend that suggests potential efficacy compared to the vehicle control (*P *= .0757). Similarly, our *in vitro* data with single-agent osimertinib are in line with existing literature on its efficacy in glioblastoma models.^38^^,^^40^^,^^41^ However, *in vivo,* osimertinib-treated mice did not differ from vehicle control mice in terms of tumor burden or survival rates. The *in vivo* model used in our study is derived from a highly aggressive GSC line characterized by loss-of-function mutations in *TP53* and *PTEN*, unmethylated MGMT, and is of a proneural molecular subtype, which results in rapid disease progression and a median survival of less than 30 days. In addition to using an aggressive *in vivo* model, we also used different mouse strains and alternative methods of implanting the GSC line orthotopically. The changes in the mouse model may contribute to differences in preclinical treatment outcomes. Although single-agent treatments did not significantly improve tumor burden or survival, the mice receiving both NCD38 and osimertinib demonstrated reduced tumor burden and increased survival compared with the vehicle control. Considering the aggressive nature of the orthotopic glioblastoma model used and the rapid disease progression, a 7-day extension represents a 25% increase in median survival. Importantly, the observed efficacy in combination treatment did not come at the cost of hematological toxicity or weight loss.

Current literature describing the interplay between epigenetic modulators, particularly LSD1, and proliferative kinase signaling pathways has consistently shown that the relationship is context-dependent and multifactorial.^46^ Studies completed in acute myeloid leukemia (AML) models have revealed a role of mTOR activity in mediating LSD1 resistance.^26^ One study found that AML lines resistant to LSD1 inhibition respond with an activation of mTOR activity as opposed to sensitive AML lines. In addition, a different study in AML found that MEK signaling could participate as a mechanism of resistance to LSD1 inhibitors.^25^ While other cancer models highlight their own unique challenges in terms of response to LSD1 inhibitors, similar complexities are observed in glioblastoma models.

Previous studies on glioblastoma have reported findings different from those observed in our study. For example, in U87MG cells, treatment with a catalytic LSD1 inhibitor, tranylcypromine, reduced mTOR activity along with mitochondrial oxidative capacity.^33^ The differences observed in this study compared to our results may be attributed to distinct mutations present in the cell lines that were used. Also, tranylcypromine differs from the LSD1 inhibitors used in our study. In particular, tranylcypromine is a nonselective monoamine oxidase inhibitor with a relatively low specificity for LSD1. Off-target effects of tranylcypromine could account for a varied response on kinase signaling pathways. In comparison to previous studies in glioblastoma, our study highlights a correlation between the activity of phospho-S6-ribosomal protein and a therapeutic response to the combination treatment of NCD38 and osimertinib. Furthermore, we observed an increase in kinase activity in association with resistance to NCD38, suggesting that resistance may be attributed to the increased kinase activity.

Our findings suggest that the combination of catalytic LSD1 inhibitors and EGFR inhibitors could be a promising therapeutic strategy and warrants continued evaluation in glioblastoma models. Our combination treatment is particularly relevant considering the complex molecular biology of glioblastoma in patients, including EGFR alterations and epigenetic dysregulation. Future studies should further elucidate the molecular mechanism of synergy from the combination treatment of LSD1 and kinase inhibition. In particular, it would be beneficial to explore the relationship between EGFR status and response to LSD1-targeted therapies. Additionally, more extensive studies with additional patient-derived models and a larger group size distribution need to be completed in various mouse models. For example, it would be important to understand the impact of the combination treatment in the context of the glioblastoma microenvironment by utilizing more immune-competent mouse models. In addition, expanding the efficacy and safety studies with larger group sizes are needed to evaluate the treatment combination, in particular, the effect on hematological parameters.

As novel strategies continue to emerge, the development of dual LSD1/EGFR inhibitors as a single molecular entity represents an attractive approach to overcoming these resistance mechanisms. Recent studies have reported the design of dual-targeted LSD1/EGFR inhibitors, demonstrating efficacy in preclinical models of non-small cell lung cancer.^47^ Given the varied effects of LSD1 inhibitors on GSC kinase activity, it remains to be determined whether dual inhibitors will be more effective than combination therapy using separate inhibitors in glioblastoma. Single-agent inhibitors may still have a role, particularly if patient- or cell-specific vulnerabilities can be identified to guide their use in tailored therapeutic strategies. Future studies should focus on optimizing dual LSD1/kinase inhibitors for glioblastoma, including high brain permeability, and understanding the factors that dictate whether a single-molecule approach or combination therapy will be more effective in overcoming resistance and improving efficacy.

## Supplementary Material

vdaf246_Supplementary_Data

## Data Availability

The data generated in this study are available upon request from the corresponding author.
