# Supplementary material for: Combined inhibition of lysine-specific demethylase 1 and kinase signaling as a preclinical treatment strategy in glioblastoma
Source: Neurooncol Adv. 2025 Nov 20;7(1):vdaf246. doi: 10.1093/noajnl/vdaf246 (PMC12768500; doi:10.1093/noajnl/vdaf246)
Supplement: vdaf246_Supplementary_Data [file vdaf246_supplementary_data.docx]

**Supplementary Data for Stitzlein et al.**

**Raw Western Images**


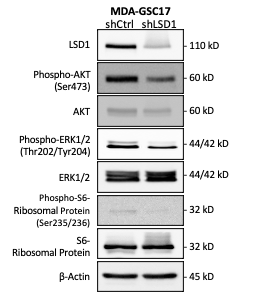
Raw western images for **Figure 1F**.


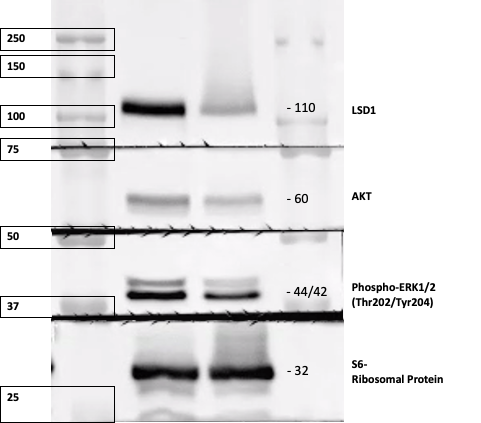

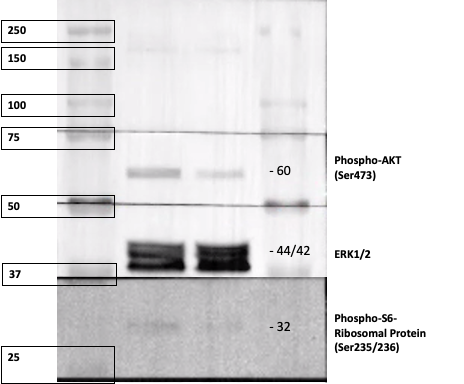


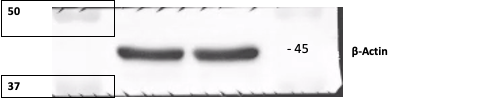


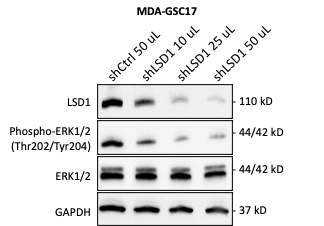

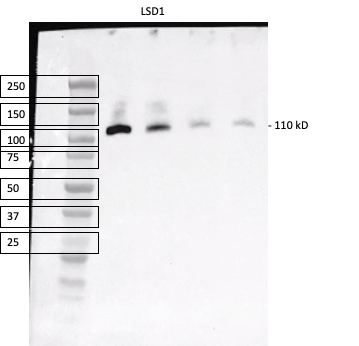
Raw western images for **Figure 1G**.


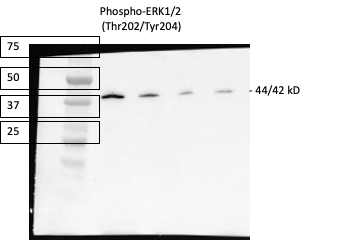


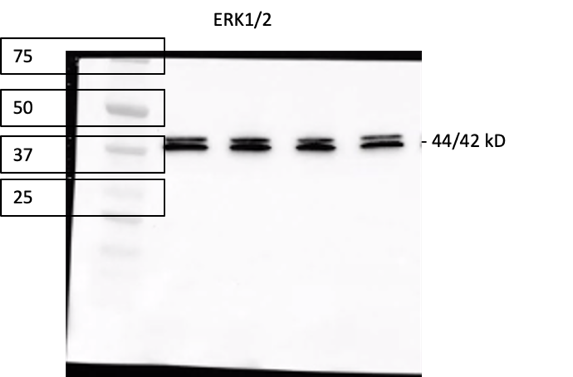


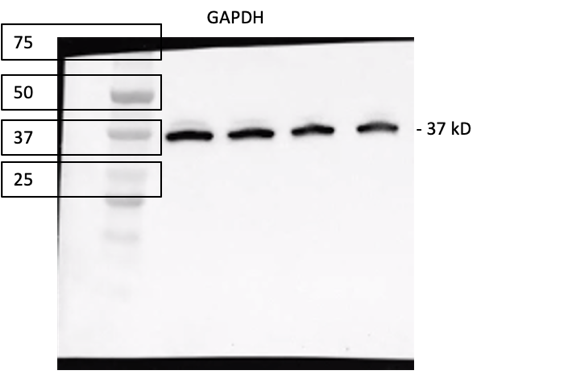


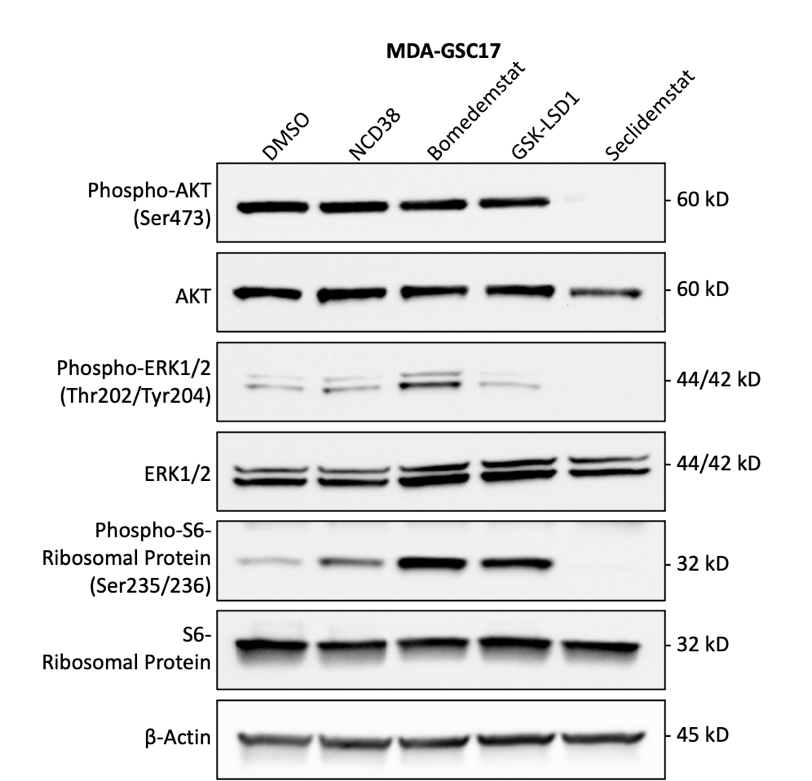
Raw western images **for Figure 1H**.


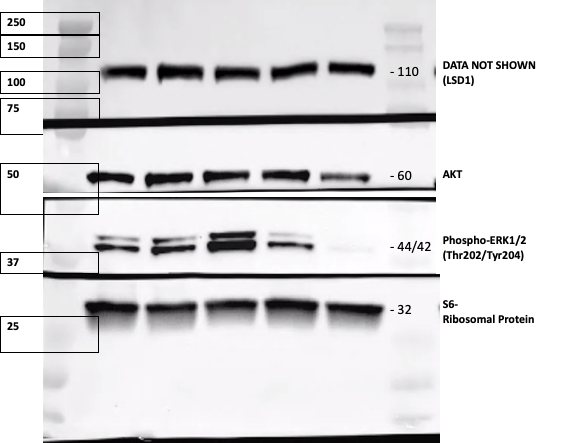

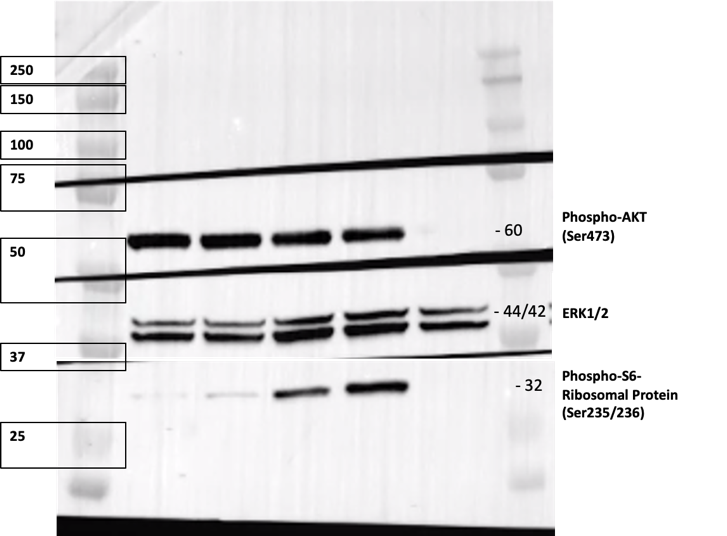


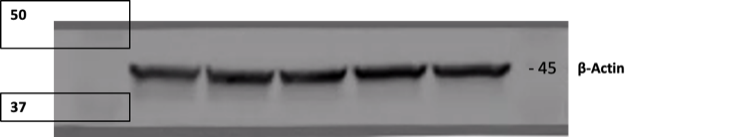


Raw western images for **Figure 1I** and **Figure 4M**.


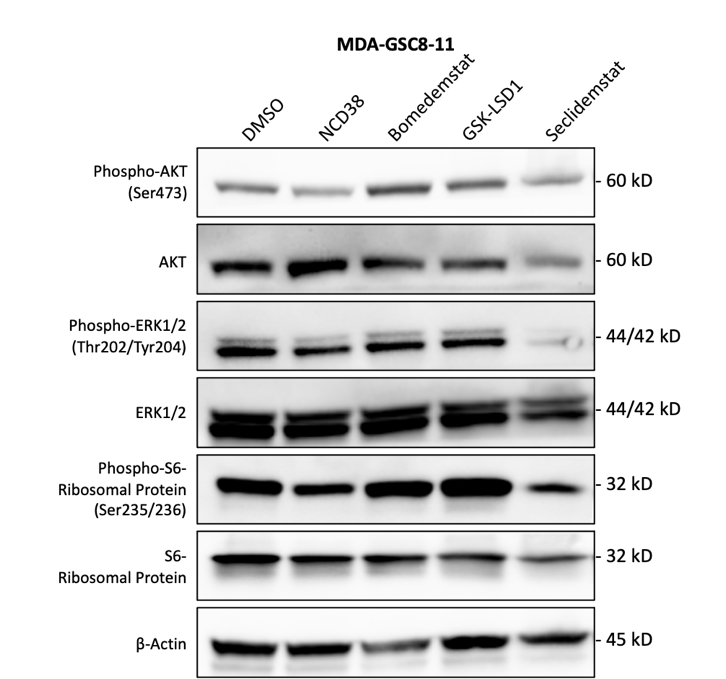


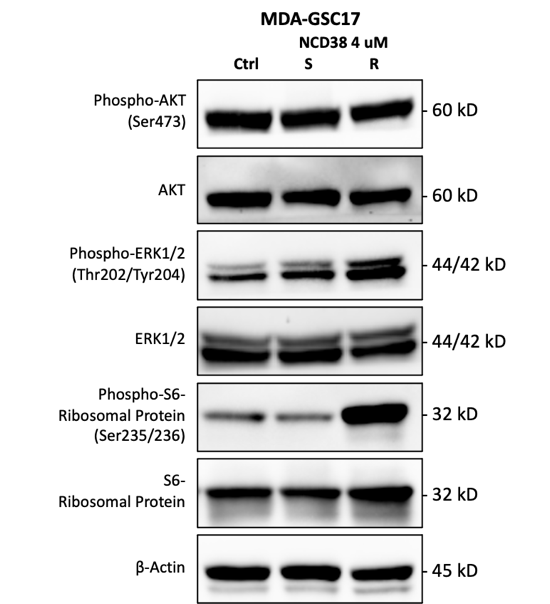


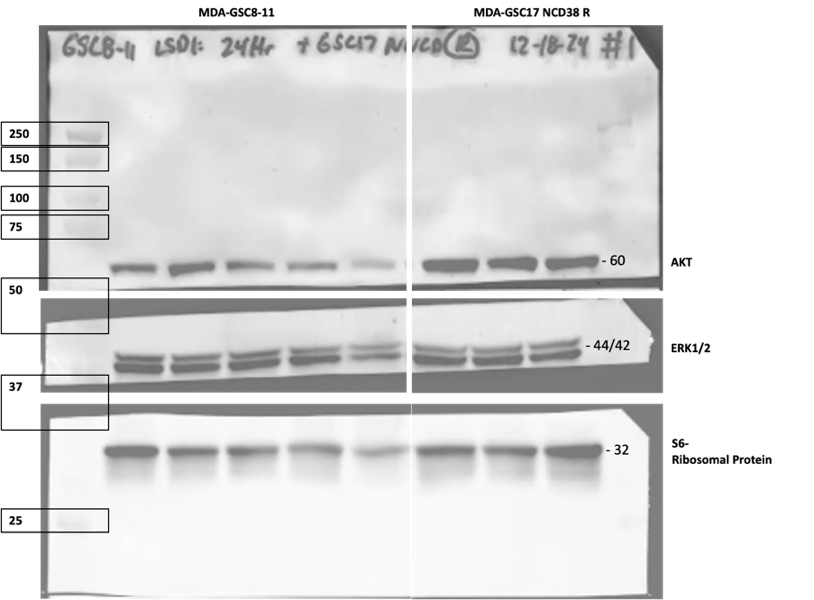

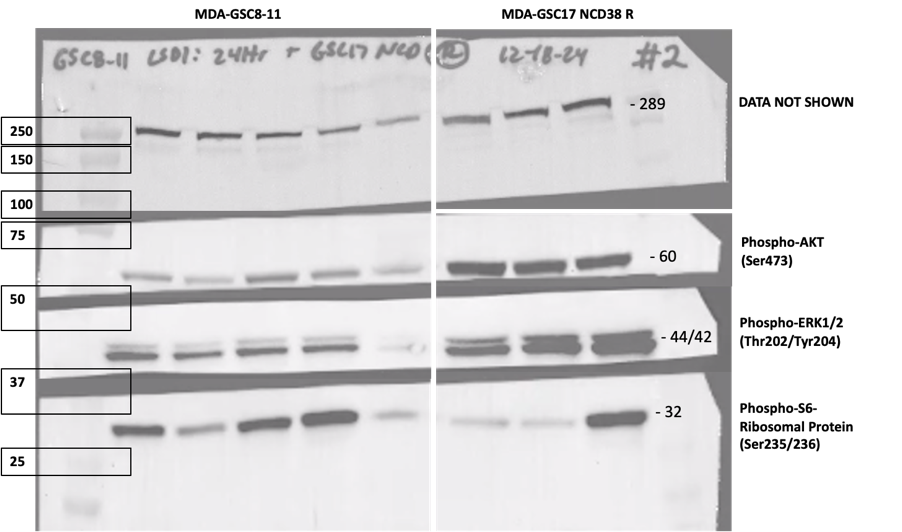


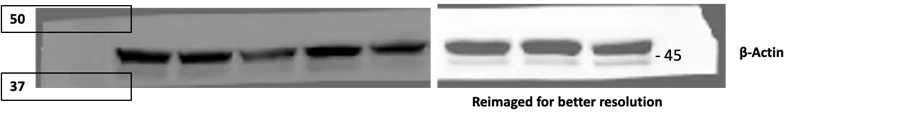


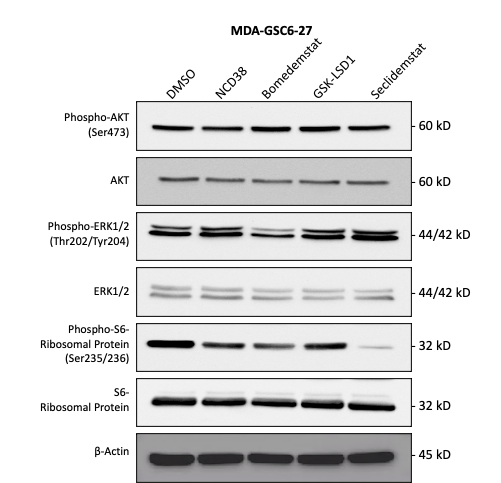
Raw western images for **Figure 1J.**


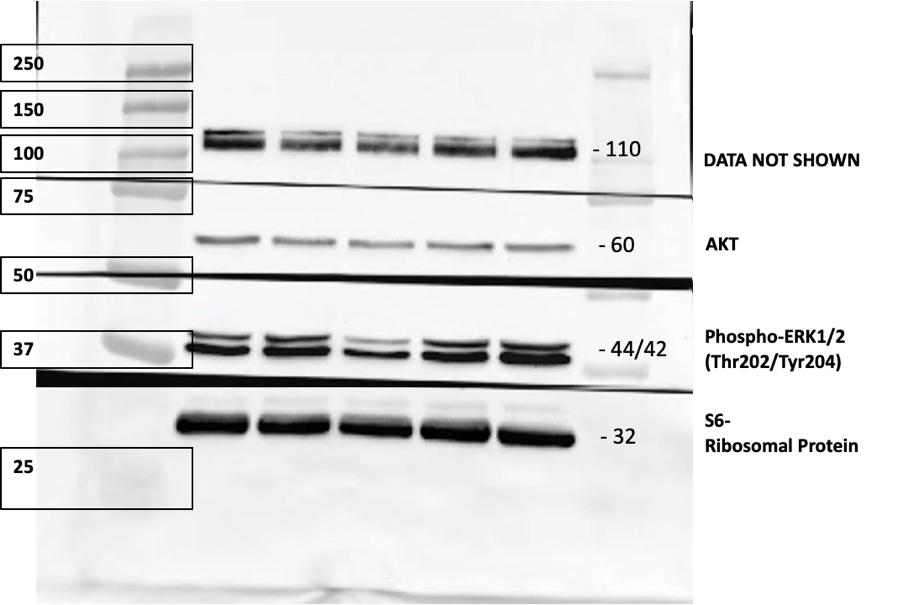

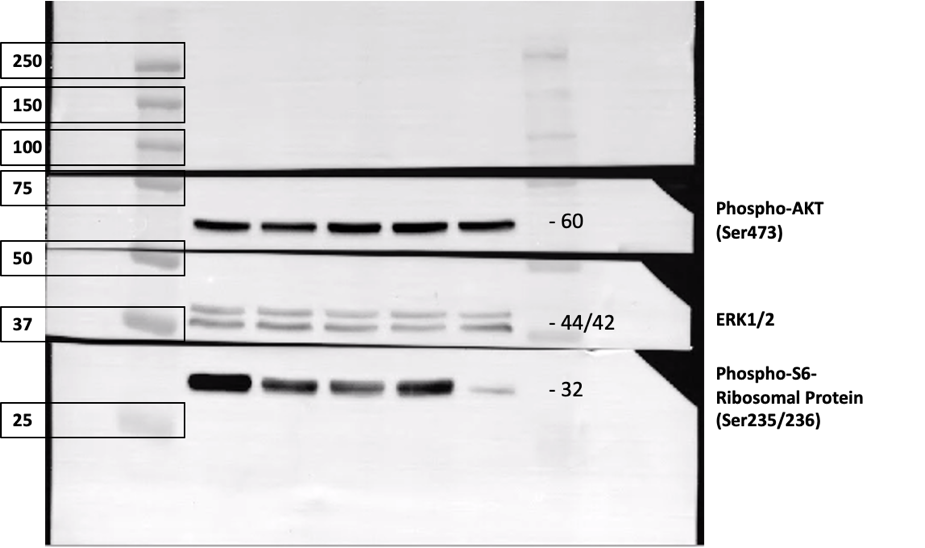


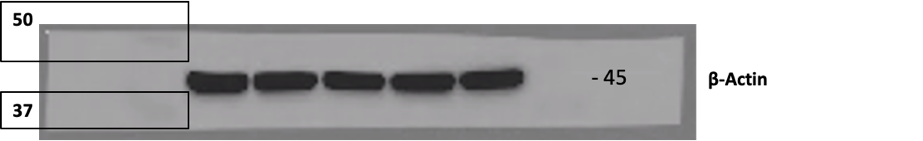


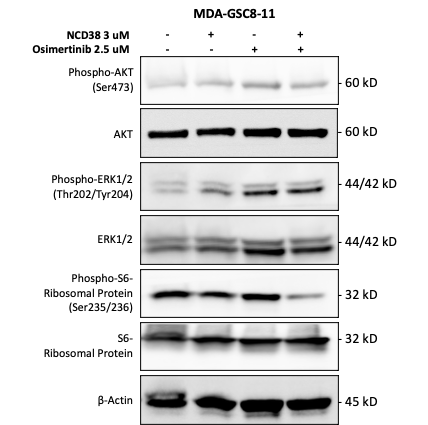

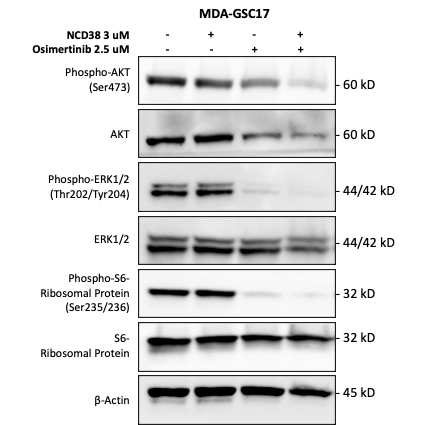
Raw western images for **Figure 4A and B**.


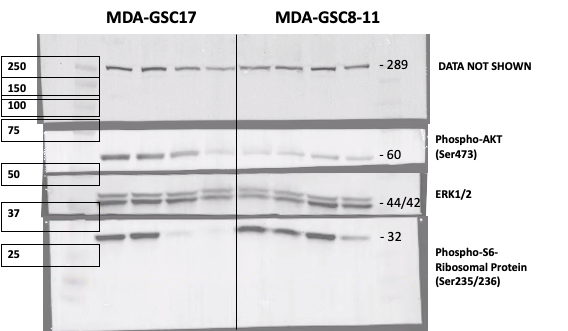


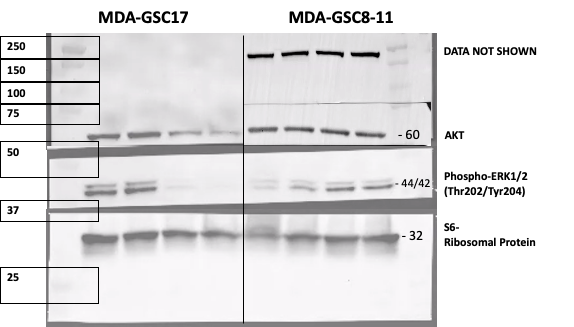


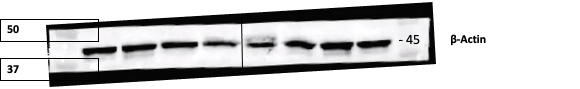


Raw western images for **Figure 4C**.


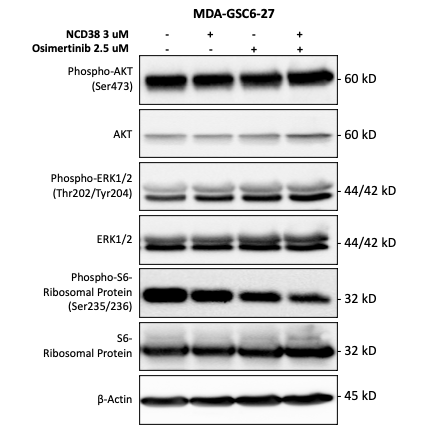


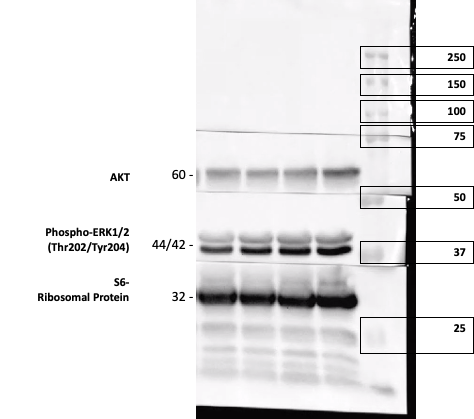

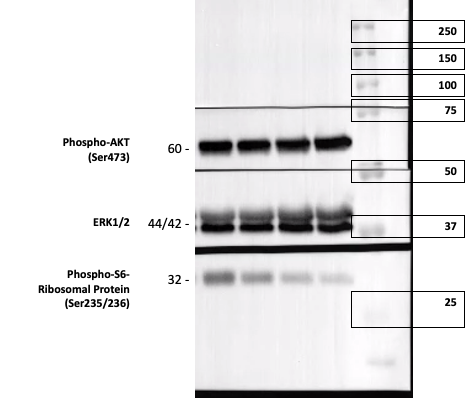


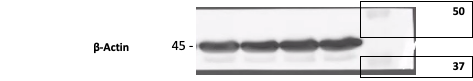


Raw western images for **Figure 4F.**


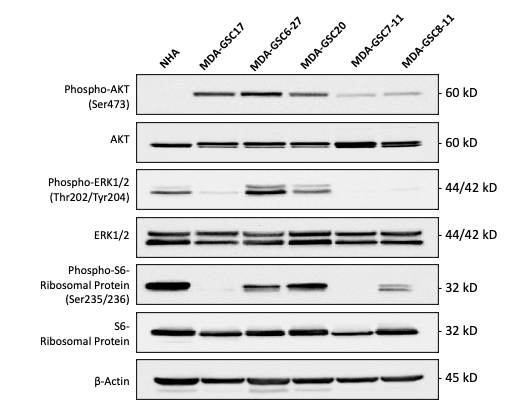


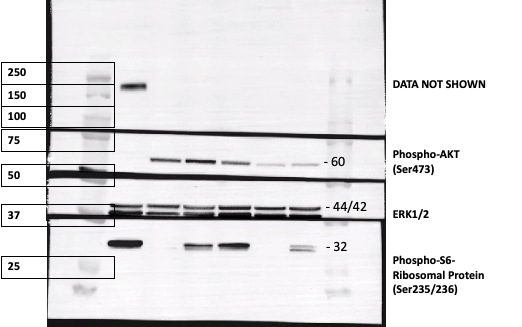


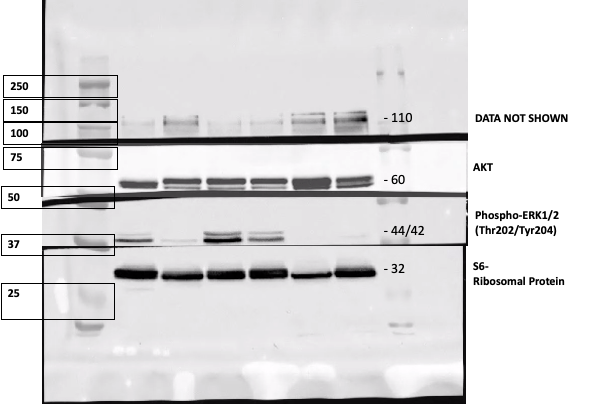


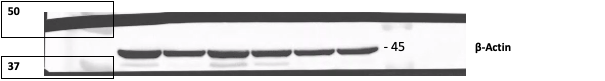


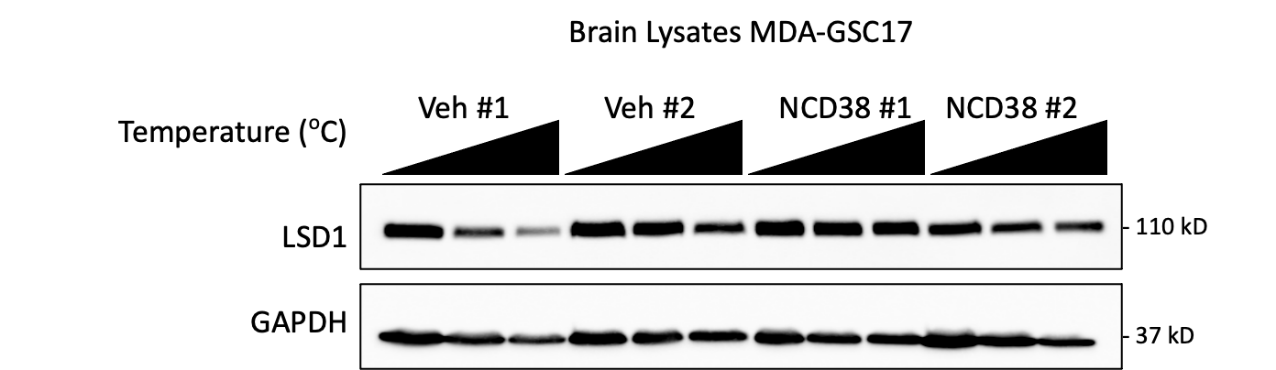
Raw western images for **Figure 5I**.


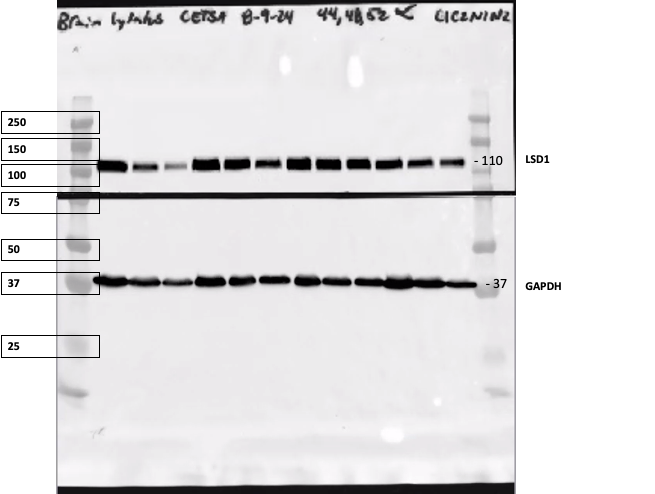


**Microscopy Images**

MDA-GSC7-11 cells treated with either DMSO (0.25%), NCD38 (1 μM), osimertinib (1.25 μM), or a combination of NCD38 (1 μM) and osimertinib (1.25 μM). In parallel with the neurosphere formation assay, the cells were observed under the microscope for changes in cell/neurosphere morphology and size across different treatment conditions.

**DMSO**

**
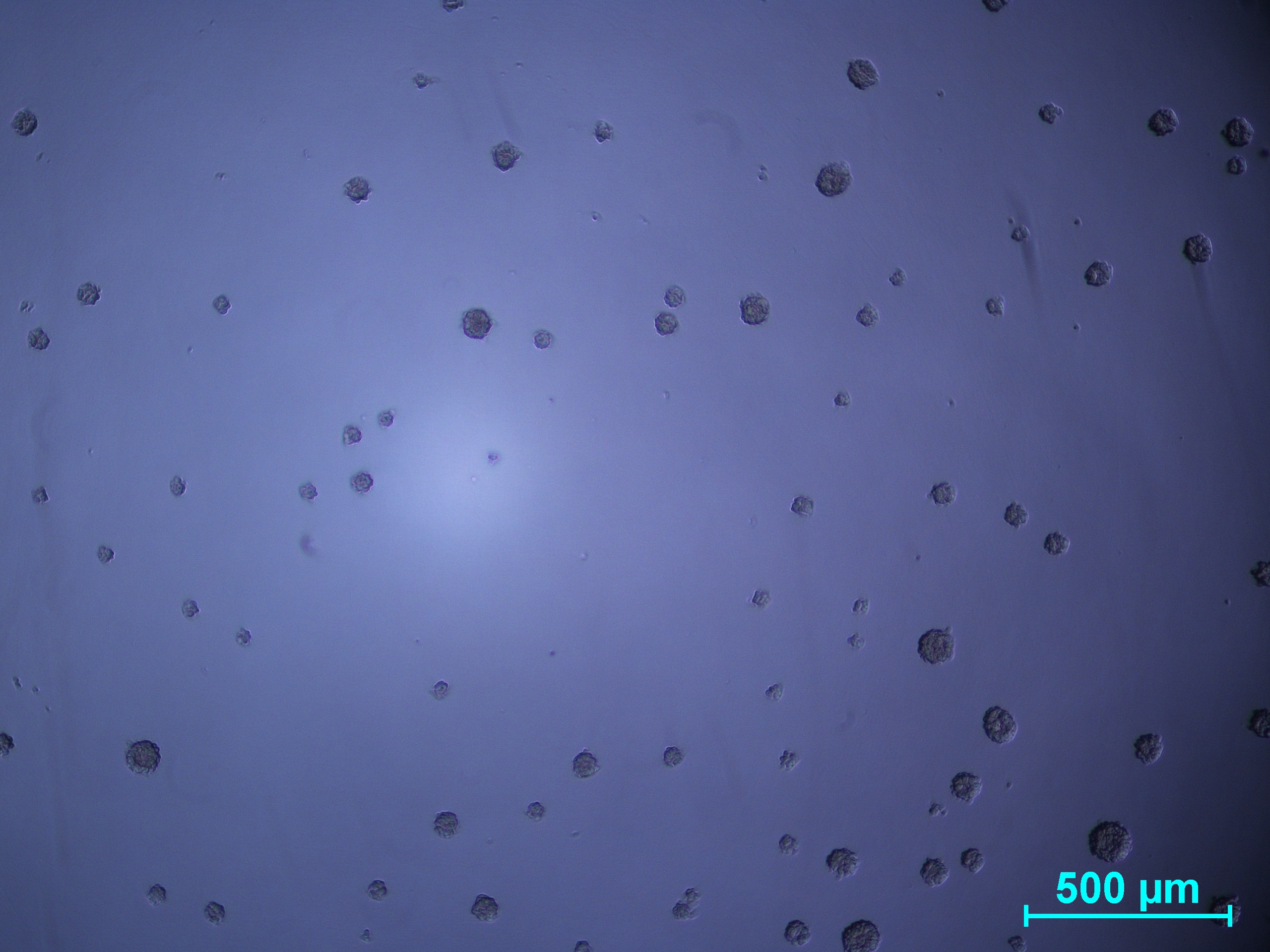
**

**NCD38 1 μM**


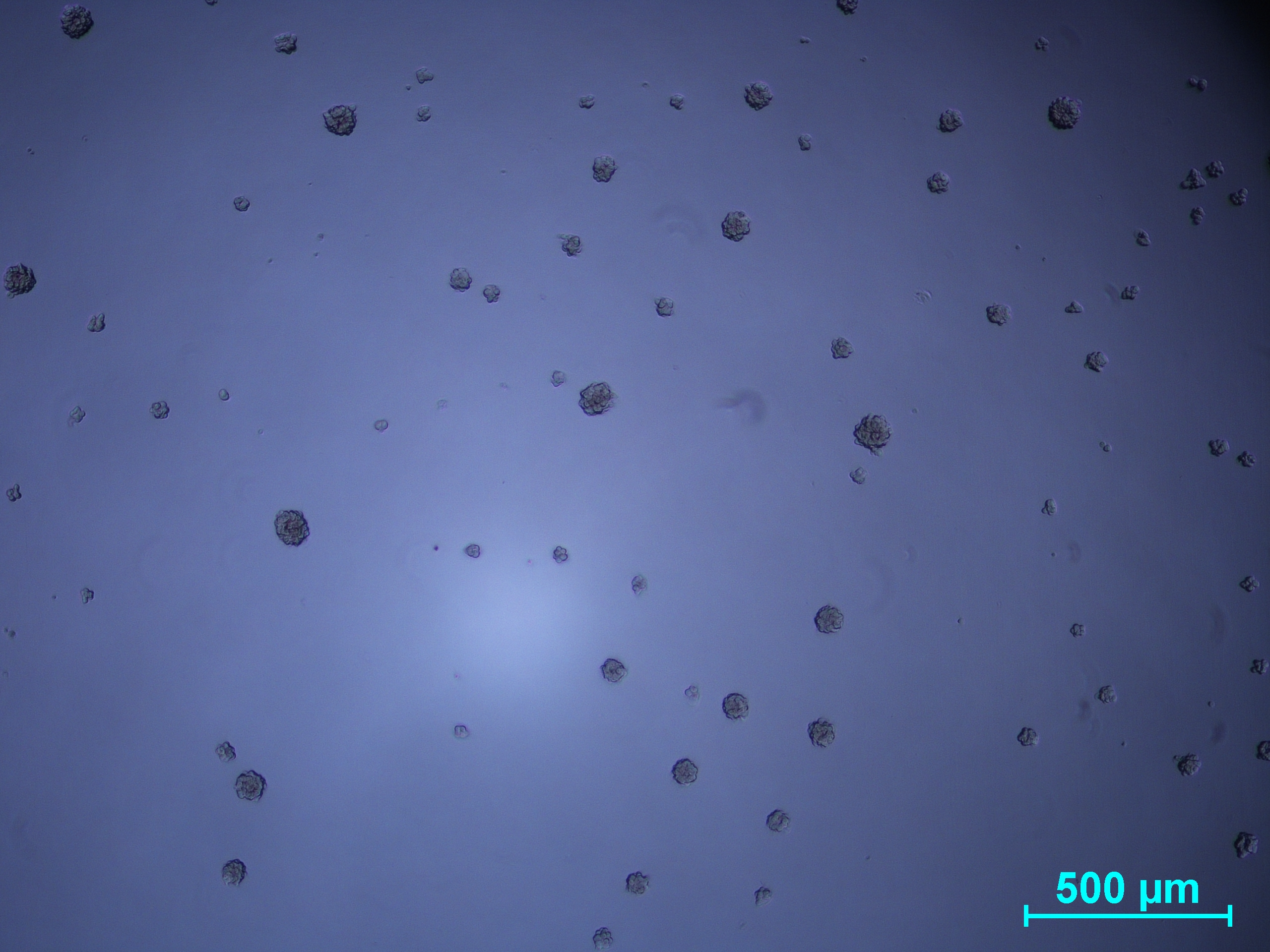


**Osimertinib 1.25 μM**

**
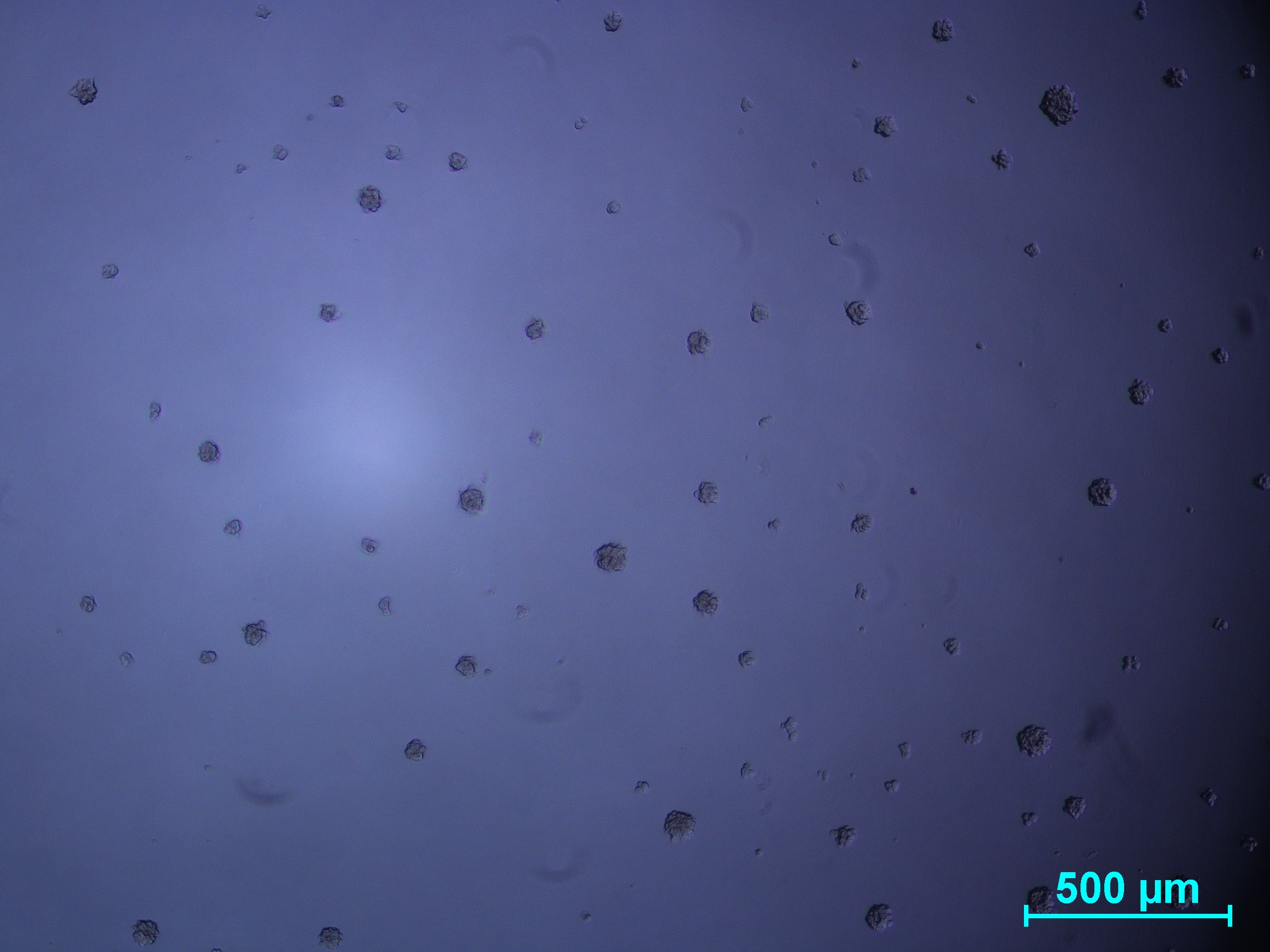
**

**NCD38 1 μM and osimertinib 1.25 μM**

**
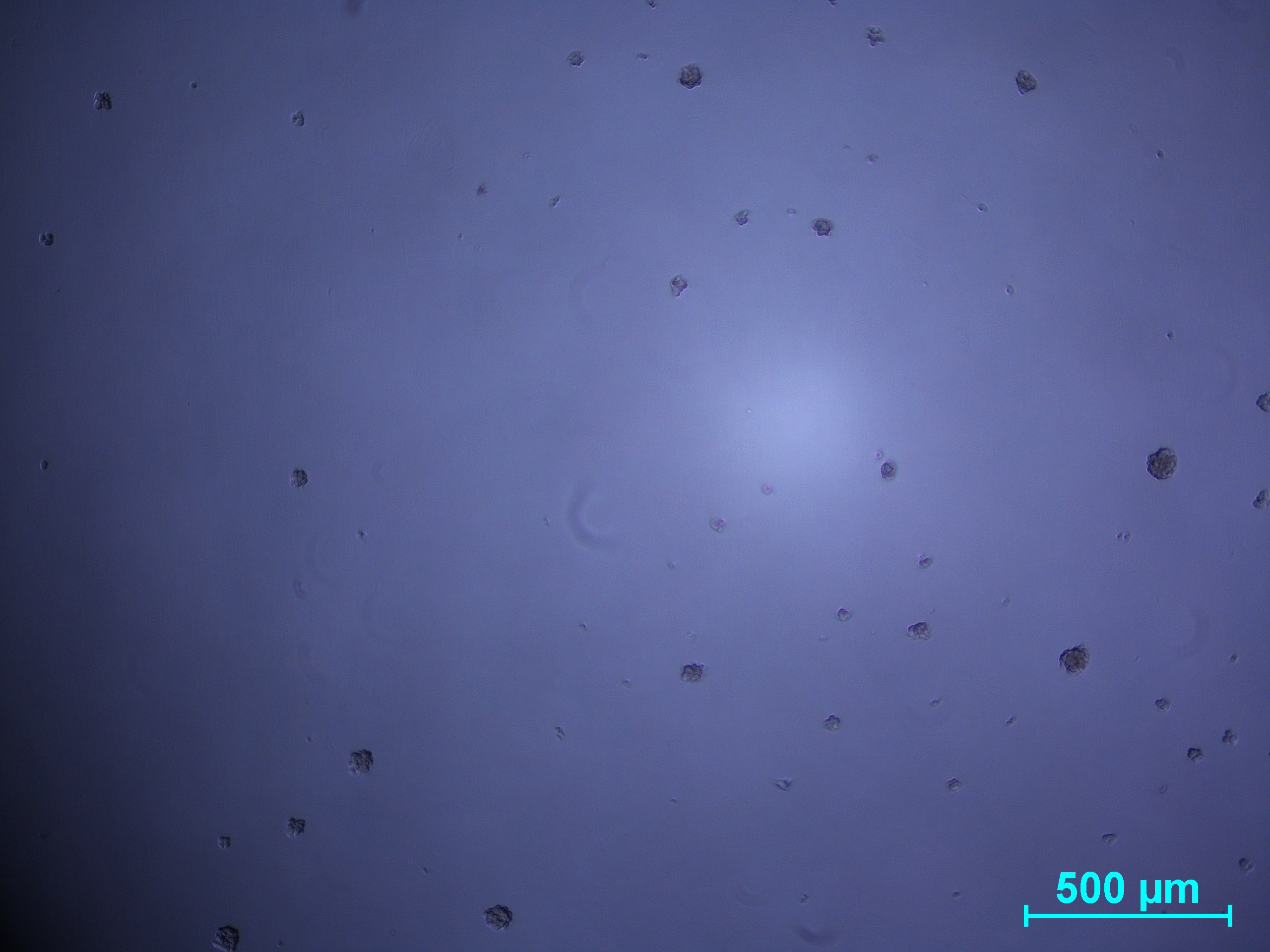
**

**
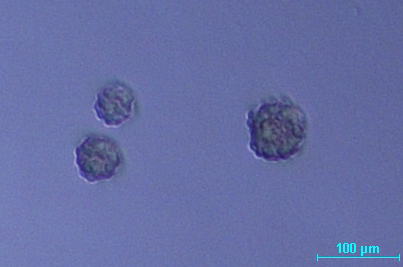
DMSO**

**NCD38 1 μM**


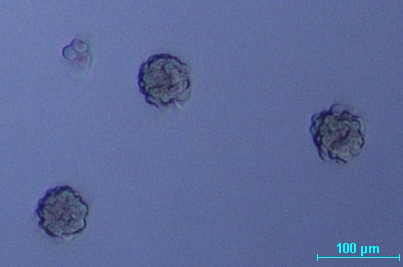


**Osimertinib 1.25 μM**


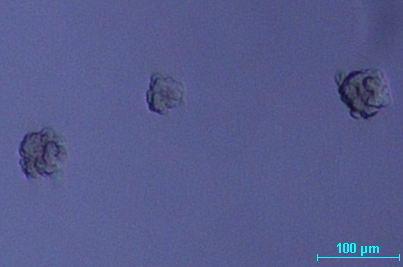


**NCD38 1 μM and osimertinib 1.25 μM**

**
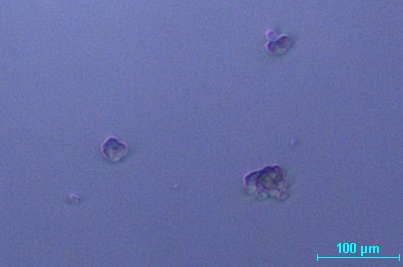
**
